# Draft Genome Sequence of *Serratia* sp. Strain DD3, Isolated from the Guts of *Daphnia magna*

**DOI:** 10.1128/genomeA.00903-14

**Published:** 2014-09-11

**Authors:** Anja Poehlein, Heike M. Freese, Rolf Daniel, Diliana D. Simeonova

**Affiliations:** a Genomic and Applied Microbiology and Göttingen Genomics Laboratory, Georg-August University, Göttingen, Germany; b Laboratory of Microbial Ecology, University of Konstanz, Constance, Germany

## Abstract

We report the draft genome sequence of *Serratia* sp. strain DD3, a gammaproteobacterium from the family *Enterobacteriaceae*. It was isolated from homogenized guts of *Daphnia magna*. The genome size is 5,274 Mb.

## GENOME ANNOUNCEMENT

*Serratia* sp. strain DD3 was isolated from homogenized gut samples of cultured *Daphnia magna*, grown in filtered oligotrophic water of Lake Constance and fed with the green alga *Scenedesmus obliquus* (1). Like most *Serratia* spp., strain DD3 is a psychrotolerant facultative lithothroph, exhibiting an anaerobic lifestyle. *Serratia* spp. are found in a variety of environments such as freshwater, insects, and vertebrates, including humans, and on plant surfaces where they act as opportunistic pathogens (2). A driver for *Serratia* pathogenicity may be phosphate starvation (3–5). The phosphorus requirements are usually covered by phosphate and phosphate esters. Under phosphate starvation some bacterial species assimilate alternatively reduced inorganic and organic P compounds (organophosphonates) (6, 7). The latter group are utilized from bacteria as P, C, and N sources (8–14).

For sequencing, genomic DNA of *Serratia* sp. DD3 was isolated with the Purgene core kit B (Qiagen, Hilden, Germany). Extracted DNA was used to prepare shotgun libraries for Illumina sequencing as recommended by the manufacturer (Illumina, San Diego, CA, USA). Sequencing resulted in 4,000,318 paired-end Illumina reads (112 bp) and a coverage of 79.94. The initial hybrid *de novo* assembly was performed with Ray (15) software and resulted in 123 contigs. The genome of *Serratia* sp. strain DD3 consists of a single circular chromosome. The overall G+C content was 49.16 mol%. YACOP and GLIMMER (16) software tools were used for automatic gene prediction, while RNAmmer and tRNAscan were used for identification of rRNA and tRNA genes, respectively (17, 18). The functional annotation of the protein-coding genes was carried out with the Intergrated Microbial Genomes/Expert Review (IMG/ER) system (19). A total of 4,666 putative genes were identified, of which 4,595 were protein encoding. Overall, 3,958 (84.83%) of the open reading frames were assigned to functions. One complete rRNA cluster out of 23 rRNA genes and 62 tRNAs, including those for selenocysteine incorporation, were identified.

Most probably, the phosphate level regulation in strain DD3 proceeds via *phoU*, located in the genome of the strain. Phosphate is delivered into the cells through a low-affinity phosphate: sulfate intake permease, a high-affinity Pst system (*pstSABC*), and a phosphate ester uptake systems (PTS), all found in the genome of the strain (20). Under phosphate starvation, strain DD3 assimilates phosphite or organophosphonates, but not hypophosphite or other reduced P compounds. It lacks the gene coding for phosphonate dehydrogenase (*ptxD*); therefore, phosphite presumably is oxidized from membrane-spanning alkaline phosphatase coupled to an [NiFe] hydrogenase, a process analogous to what occurs in *Escherichia coli* (13). The ability of DD3 to grow with phosphite as the single phosphorus source was proven with physiological experiments (D. D. Simeonova, unpublished data). Organophosphonate uptake and assimilation pathways identified were the 2-aminoethylphosphonate (2AEP) assimilation phosphonoacetaldehyde hydrolase-dependent (PhnWX) pathway (11), the phosphonoacetate hydrolase (PhnA) pathway (21, 22), and a complete C-P lyase operon (23), which allows the utilization of the broadest spectrum of organophosphonates, and phosphite.

### Nucleotide sequence accession numbers.

This whole-genome shotgun project has been deposited at DDBJ/EMBL/GenBank under the accession number AYKS00000000. The version described in this paper is the second version, AYKS20000000.
